# The effects of increased acetate turnover on glucose-induced insulin secretion in lean and obese humans

**DOI:** 10.1017/cts.2018.342

**Published:** 2019-06-17

**Authors:** Kitt Falk Petersen, Anne Impellizeri, Gary W. Cline, Gerald I. Shulman

**Affiliations:** 1 Department of Internal Medicine, Yale University School of Medicine, New Haven, CT, USA; 2 Novo Nordisk Foundation Center for Basic Metabolic Research, University of Copenhagen, Copenhagen, Denmark; 3 Department of Cellular and Molecular Physiology, Yale University School of Medicine, New Haven, CT, USA

**Keywords:** Acetate turnover, insulin secretion, ghrelin, obesity, hyperglycemia

## Abstract

**Introduction:**

Increased endogenous acetate production (Ra) in rodents has been shown to activate the parasympathetic nervous system and thereby promote increased glucose-stimulated insulin secretion (GSIS), increased ghrelin secretion, hyperphagia and obesity.

**Aim:**

To examine whether rates of acetate turnover are different in lean versus obese humans and whether increased acetate turnover promotes increased GSIS and increased ghrelin secretion in humans.

**Methods:**

Basal acetate Ra was measured following an overnight fast in lean (BMI: 21.3 ± 1.1 Kg/m^2^) and obese (30.2 ± 0.9 Kg/m^2^, *P* = 0.00001) individuals. The subjects underwent two hyperglycemic (10 mmol/L) clamp studies to measure GSIS during a basal acetate infusion and during a high-dose acetate infusion increasing plasma acetate concentrations ∼5-fold.

**Results:**

Basal acetate Ra was 30% higher in the lean compared to the obese subjects (257 ± 27 vs. 173 ± 18 μmol/min; *P* = 0.025). Basal plasma insulin concentrations were 4-fold higher in the obese than the lean subjects (*P* = 0.008) and increased 5-fold during hyperglycemia in both groups, independent of changes in plasma acetate concentrations. Fasting plasma ghrelin concentrations were 35% lower in the obese compared to the lean subjects (*P* = 0.015). During the hyperglycemic clamp, plasma ghrelin decreased by 42% in the lean group (*P* < 0.022 vs. basal) and did not change in the obese group.

**Conclusion:**

Rates of endogenous acetate turnover are ∼30% higher in the lean subjects compared to the obese subjects, and increasing plasma acetate turnover does not promote increased GSIS or ghrelin secretion in either group.

## Introduction

The potential impact of acetate and gut microbiota on appetite regulation and body weight has been studied extensively in rodent models, but these studies have led to differing conclusions [1–3]. Perry *et al.* assessed rates of endogenous acetate turnover in lean regular chow and high-fat-fed (HFF) obese rats and found that high-fat feeding promoted increased rates of endogenous acetate turnover due to increased acetate production by the gut microbiota [3]. Consistent with these findings, studies in humans have found increased fecal acetate concentrations in obese individuals [4]. Perry *et al.* went on to demonstrate that when acetate is infused in physiological amounts, to match endogenous rates of acetate turnover in HFF obese rats, this perturbation led to activation of the parasympathetic nervous system, which in turn promoted increased glucose-stimulated insulin secretion (GSIS), increased ghrelin secretion, hyperphagia and obesity [3]. In contrast, supra-physiological doses of acetate in rodents have been shown to suppress appetite and lead to weight loss and/or reduced weight gain [1, 2].

Acetate metabolism in humans has been less well studied and it is not known if plasma concentrations and/or rates of endogenous acetate Ra are altered in obese versus lean humans. Furthermore, it is not known if alterations in plasma acetate concentrations can impact GSIS in humans, as it has been shown to do in rodents. Given the prior results in rodents, we hypothesized that: (1) whole body acetate turnover would be increased in obese humans compared to lean humans and (2) increased acetate turnover would promote increased GSIS and ghrelin secretion. In order to examine these hypotheses, we assessed rates of basal whole body acetate turnover in lean and obese humans using an infusion [2-^13^C]acetate. In order to examine the effects of increased plasma acetate concentrations on GSIS and ghrelin secretion, the subjects underwent two hyperglycemic clamps combined with a tracer infusion of [2-^13^C]acetate and a high-dose infusion of [2-^13^C]acetate designed to increase acetate turnover by ∼5-fold and raise plasma acetate concentrations ∼5-fold.

## Materials and Methods

Six healthy obese (age: 46 ± 6 years, BMI: 30.2 ± 0.8 Kg/m^2^) and six healthy lean (age: 39 ± 5 years, BMI: 21.3 ± 1.1 Kg/m^2^) individuals were recruited from the greater New Haven district. All had normal glucose tolerance, as verified by a 3-h 75-g oral glucose tolerance test (OGTT), a sedentary lifestyle and no participation in any regular exercise regimens. This was confirmed by a questionnaire and three consecutive days of activity monitoring using pedometers (GO-Walking; Sportline, Hazleton, PA) [5]. Insulin sensitivity was assessed from the OGTT using the Insulin Sensitivity Index [5, 6]. Body composition was measured by bioelectrical impedance (Tanita BC-418; Tanita, Arlington Heights, IL). Liver, intramyocellular and extramyocellular lipid contents were measured using ^1^H magnetic resonance spectroscopy (MRS) at 4 Tesla as previously described (Science 2003). All subject data are shown in Table 1.

**Table 1.** Plasma concentrations of total and acylated ghrelin in the fasting state and during hyperglycemic conditions with a basal (0.7 μmol/Kg-min) and with a high-dose infusion of acetate (14 μmol/Kg-min) in healthy lean (*n* = 6) and obese (*n* = 6) individuals

Plasma total ghrelin concentrations (pmol/L)	Lean (*n* = 6)	Obese (*n* = 6)	*P*-value
Basal	189 ± 20	124 ± 11	0.015
Glucose-stimulated/basal acetate	178 ± 10	100 ± 4	0.00004
Glucose-stimulated/high-dose acetate	133 ± 4	104 ± 9	0.011
*P*-value (Glucose-stimulated basal vs. high-dose acetate)	0.022	0.09	
**Plasma acylated ghrelin concentrations (pmol/L)**			
Basal	78 ± 9	43 ± 5	0.006
Glucose-stimulated/basal acetate	63 ± 5	38 ± 1	0.0007
Glucose-stimulated/high-dose acetate	57 ± 5	42 ± 3	0.016
*P*-value (Glucose-stimulated basal vs. high-dose acetate)	0.075	0.78	

Subjects were admitted to the Yale Center for Clinical Investigation, Hospital Research Unit (HRU) at Yale-New Haven Hospital on two separate occasions and studied at 0800 following a 12-h overnight fast. The 12 subjects participated in two studies, which were performed in a randomized order. Each study consisted of insertion of an intravenous line in an antecubital vein followed by a 2-h baseline tracer infusion of [2-^13^C]acetate (99% atom percent enrichment, 350 mmol/L sodium salt, Cambridge Isotope Laboratories Inc.) at a rate of 0.7 μmol/(Kg-min) to assess basal rates of acetate Ra. After 90 min, a retrograde intravenous line was placed in a hand vein for blood collections and the hand was placed in a warming box for the duration of the study in order to ‘arterialize’ the blood. After 120 min, baseline blood samples were collected and a 3-h hyperglycemic clamp was begun with a primed, variable infusion of dextrose (20 g/100 mL) increasing plasma glucose concentrations to ∼10 mmol/L to assess GSIS and ghrelin secretion. During one of the studies, the baseline tracer infusion of [2-^13^C]acetate at a rate of 0.7 μmol/(Kg-min) was maintained throughout the 3-h hyperglycemic clamp to maintain basal plasma acetate concentrations. During the other study, the impact of increased plasma acetate concentrations on GSIS and ghrelin secretion was examined during the hyperglycemic clamp by increasing the infusion of [2-^13^C]acetate to 14 μmol/(Kg-min) to increase plasma acetate concentrations 4–5 fold. Rates of energy expenditure and the respiratory quotient (RQ) were measured by 30 min of indirect calorimetry during both the baseline and the hyperglycemic periods (Deltatrac; Datex-Ohmeda, Madison, WI).

### Ethics Statement

The study was approved by the Yale University Human Investigational Committee, informed, written consent was obtained and the studies were conducted in compliance with the Declaration of Helsinki.

### Statistical Analyses

Sample size calculations were based on the differences in whole body acetate Ra we observed between control and high-fat-diet-fed rodents with a difference between groups of at least 50% [3]. Statistical power analysis indicated that five individuals per group were required to have 80% power to detect >50% difference in acetate turnover through the use of a two-sided un-paired *t*-test and a significance alpha level of 0.05. We, therefore, included six subjects per group.

Statistical analyses were performed with StatPlus software. Comparisons between the groups were performed using un-paired Student’s *t*-tests and comparisons within groups were performed using a paired *t*-test. *P*-values <0.05 were considered statistically significant. All data are expressed as mean ± SEM.

## Results

The BMI was significantly higher in the obese than the lean individuals (*P* < 0.0001). Following an overnight fast, plasma acetate concentrations were similar in the lean (26 ± 3 μM) and obese (32 ± 6 μM, *P* = 0.38) subjects, whereas rates of acetate turnover (Ra) were 30% higher in the lean subjects (257 ± 27 μmol/min) compared to the obese subjects (173 ± 18 μmol/min; *P* = 0.025).

During the hyperglycemic clamp and tracer acetate infusion, plasma acetate concentrations remained constant at basal fasting concentrations. Similarly, during the hyperglycemic clamp and tracer acetate infusion studies, rates of acetate Ra remained unchanged in both groups and remained ∼30% lower in the obese subjects (144 ± 14 μmol/min) than in the lean subjects (196 ± 14 μmol/min; *P* = 0.023, *lean vs. obese*).

During the combined hyperglycemic clamp and high-dose acetate infusion, plasma acetate concentrations increased similarly in the two groups (lean 188 ± 41 μmol/L; *P* = 0.009 vs. basal acetate concentrations, obese: 301 ± 52 μmol/L; *P* = 0.0035 vs. basal acetate concentrations, *P* = 0.120, lean vs. obese).

**Fig. 1.**
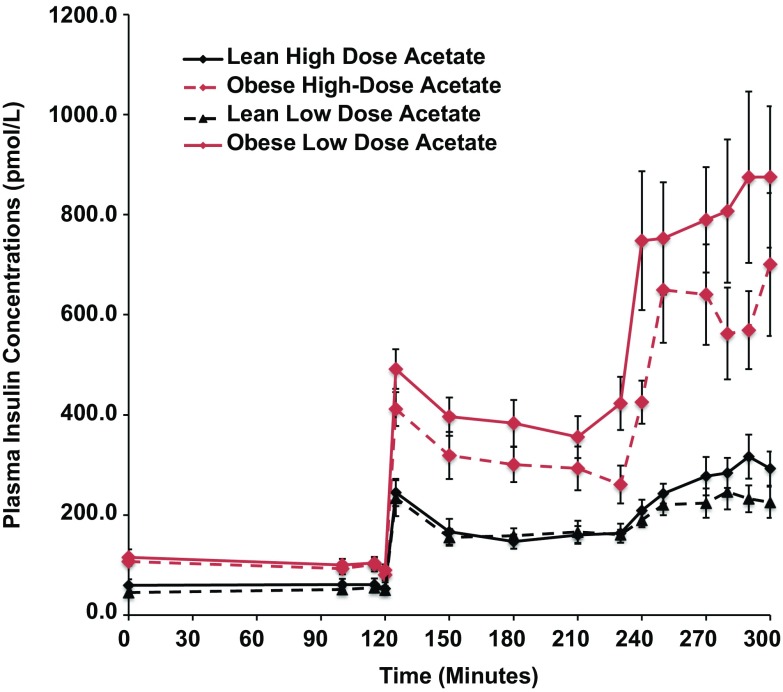
Effects of hyperglycemia on plasma insulin concentrations in lean (*n* = 6) and obese (*n* = 6) individuals during a hyperglycemic (10 mmol/L) clamp when plasma acetate concentrations were kept at basal with an acetate infusion of 0.7 μmol/Kg-min and during a high-dose (14 μmol/Kg-min) acetate infusion increasing plasma acetate concentrations ∼5-fold.

Basal plasma insulin concentrations were 4-fold higher in the obese than the lean subjects (*P* = 0.008) and increased 5-fold in both groups during hyperglycemia, independent of changes in plasma acetate concentrations (Fig. 1).

Fasting total and acylated plasma ghrelin concentrations were 35% lower in the obese group compared to the lean group (*P* = 0.015) (Table 1). During the hyperglycemic clamp tracer acetate infusion studies, total and acylated ghrelin concentrations remained unchanged in both groups (data not shown). During the hyperglycemic clamp high-dose acetate infusion studies, the concentrations of both total and acylated ghrelin decreased by 42% in the lean group (*P* = 0.022 vs. basal) and remained unchanged in the obese group (Table 1).

## Discussion

In humans, acetate has been shown to cross the blood–brain barrier [7], and increased plasma acetate concentrations have been associated with increased subjective satiety ratings [8] and reduction in body weight [9]; however, no studies have directly examined whether acetate turnover is increased in obese vs. lean humans and whether GSIS is affected by altering plasma acetate concentrations. Here, we show that plasma acetate concentrations were similar in healthy lean and obese subjects and that acetate turnover was surprisingly decreased by 30% in the obese subjects compared to the lean subjects.

In addition, we examined the effects of increased plasma acetate concentrations on GSIS and ghrelin secretion and found that while GSIS was markedly increased in the obese subjects compared to the lean subjects, there was no effect of increased plasma acetate concentrations on GSIS or ghrelin secretion in the obese subjects. In contrast, increased plasma acetate concentrations caused a decrease in total ghrelin concentrations and showed a strong tendency to decrease acylated ghrelin concentrations during the hyperglycemic clamp in the lean subjects.

These results are in marked contrast to recent studies in awake lean and obese rodents that found that increased acetate production by the gut microbiota, in response to increased caloric intake, leads to increased parasympathetic activity, which in turn promotes increased GSIS and ghrelin secretion [3]. The results were supported by additional studies showing that an acute infusion of acetate to match endogenous rates of acetate turnover in the HHF obese rats also increased GSIS and ghrelin secretion [3].

Taken together, these results demonstrate important species differences in the effects of acetate turnover on GSIS and ghrelin secretion between humans and rodents. Given the numerous studies demonstrating that alterations in the gut microbiota can impact glucose metabolism and weight gain in rodents and the key role that gut microbiota-derived acetate has on GSIS and ghrelin secretion in rats and mice, these data provide a cautionary note in translating the impact of alterations in gut microbiota and acetate turnover on intermediary metabolism and feeding behavior in rodents to humans.
